# Chromosome-Level Genome Assembly and Annotation of the Chinese Lizard Gudgeon (*Saurogobio dabryi*)

**DOI:** 10.3390/ani16172647

**Published:** 2026-08-24

**Authors:** Lei Fang, Xin Liu, Yiming Huang, Kun Yang, Yongkang Jiang, Chiping Kong, Guiqin Zou, Cheng Qian, Yutong Zhou, Lie Cao, Daxian Zhao, Wanchang Zhang

**Affiliations:** 1 Jiujiang Academy of Agricultural Sciences, Jiujiang 332005, China; 2 School of Life Sciences, Nanchang University, Nanchang 330031, China; 3 Key Laboratory of Aquaculture Germplasm Innovation and Utilization of Jiangxi Province, Nanchang 330031, China; 4 Wuning County Bureau of Agriculture and Rural Affairs, Jiujiang 332399, China; 5 Lushan City Bureau of Agriculture and Rural Affairs, Jiujiang 332800, China; 6 Pengze Crucian Carp Fine Breed Farm, Pengze County, Jiujiang 332701, China; 7 Pengze County Bureau of Agriculture and Rural Affairs, Jiujiang 332701, China

**Keywords:** *Saurogobio dabryi*, genome assembly, genome annotation, benthic adaptation, phylogenetic relationship, aquaculture

## Abstract

The Chinese lizard gudgeon (*Saurogobio dabryi*) is an economically important freshwater species within the Cyprinidae family, abundant in the middle and lower reaches of the Yangtze River and its adjacent basins. As a promising species suitable for aquaculture in China, the lack of genomic resources has rendered the genetic breeding and conservation research. Here, we present the first chromosome-level genome assembly of *S. dabryi* using PacBio HiFi long reads, short reads, and Hi-C sequencing data. The final assembly reaches a total size of 1.09 Gb and Hi-C scaffolding anchors 99.55% of the assembled contigs onto 25 chromosomes, with a scaffold N50 reaching 43.15 Mb. The final genome assembly shows a BUSCO completeness of 98.39%. We annotated 659.55 Mb repetitive sequences and 26,036 protein-coding genes, 99.47% of which are functionally annotated. Comparative phylogenomic analysis clarifies the phylogenetic position of *Saurogobio* within Gobioninae. This high-quality genome provides a critical genetic basis for exploring cyprinid phylogeny, benthic adaptive evolution, genetic improvement, and conservation efforts of *S. dabryi*.

## Simple Summary

The Chinese lizard gudgeon (*Saurogobio dabryi*) is a commercially important benthic freshwater fish endemic to East Asian river basins, with high ecological and aquaculture value. However, the lack of a high-quality reference genome has limited research on its adaptive evolution, genetic breeding, and wild population conservation. In this study, we constructed the first chromosome-level genome assembly of *S. dabryi* by integrating PacBio HiFi long-read sequencing, short-read sequencing, and Hi-C chromatin interaction technology. The final assembly spans 1.09 Gb with 25 chromosomes, and exhibits high continuity, completeness, and accuracy. We further annotated repetitive sequences, protein-coding genes, and non-coding RNAs, and explored its phylogenetic position within Cyprinidae. This genomic resource provides a fundamental basis for future studies on benthic fish adaptation, germplasm improvement, and biodiversity conservation of Gobioninae species.

## 1. Introduction

**Figure 1:**
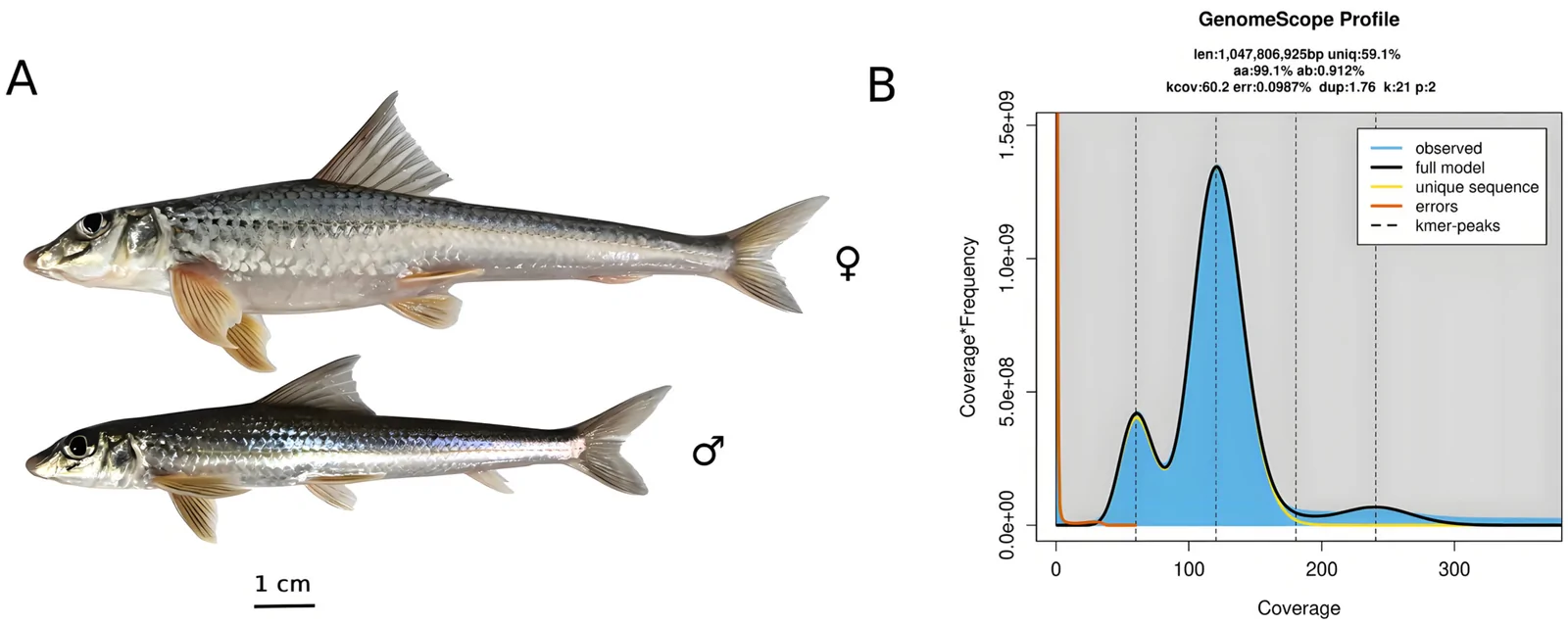
Morphology and genome *k*-mer analysis of the Chinese lizard gudgeon (*Saurogobio dabryi*). (**A**) Morphological photographs of female (**upper**) and male (**lower**) individuals, scale bar equals 1 cm. (**B**) GenomeScope *k*-mer frequency profile based on 21-mer for genome size estimation. The light blue area represents observed k-mer coverage frequency; the black line denotes the full fitted model; the yellow line shows unique sequence distribution; the orange line indicates sequencing errors; and dashed vertical lines mark characteristic k-mer peaks. Top text labels summarize core genomic metrics: total genome length, unique sequence proportion, heterozygosity (ab), duplication ratio (dup), k-mer size (k), and ploidy (p).

Gobioninae is a diverse subfamily of benthic freshwater cyprinids endemic to East Asian rivers. These fishes exhibit specialized adaptations to lotic habitats and serve as valuable models for studying aquatic adaptation, anthropogenic population differentiation, and freshwater conservation. The Chinese lizard gudgeon (*Saurogobio dabryi*, Figure 1A), a typical benthic gobionid, is widely distributed across the Yangtze, Yellow, and Pearl River basins of China [1,2]. This small demersal species inhabits sandy and silty riverbeds, acting as a key omnivorous detritivore and secondary consumer in fluvial food webs and a reliable bioindicator of river ecosystem health [3]. In addition to its ecological importance, *S. dabryi* has emerged as a commercially valuable aquaculture species in China due to its delicate flesh and unique flavor, alongside other common benthic cyprinids including *Pseudorasbora parva* [4], *Coreius heterodon* [5], and *Sarcocheilichthys sinensis* [6].

Until now, multiple studies have documented key biological characteristics of *S. dabryi*, including hydropower-driven population differentiation [7,8], morphological sexual dimorphism [9], feeding habits [10], and wild population dynamics [11]. However, available genetic evidence relies predominantly on short mitochondrial markers (e.g., *Cyt b*) and limited nuclear loci [11,12], which only resolve coarse-scale population genetic structure. The absence of a high-quality chromosome-level reference genome has severely hindered in-depth research. Furthermore, the phylogenetic placement of the genus *Saurogobio* within Gobioninae and its genome-level evolutionary divergence from other benthic cyprinids have not been systematically clarified.

In this study, we constructed the first chromosome-level reference genome of *S. dabryi* by integrating PacBio HiFi long reads, whole-genome short reads, and Hi-C chromatin interaction data. Multi-tissue transcriptomic sequencing was used to support accurate gene structure prediction and comprehensive functional annotation. We evaluated genome continuity, completeness, and accuracy through multiple orthogonal approaches. We further explored the phylogenetic relationship between *S. dabryi* and other benthic cyprinids. This high-quality chromosome-level assembly provides a fundamental genomic resource for future studies on benthic fish adaptive evolution, population differentiation, and sex dimorphism, and supports the conservation, restoration, and sustainable management of wild *S. dabryi* populations in human-regulated river ecosystems.

## 2. Materials and Methods

### 2.1. Sample Collection and Ethical Approval

Adult healthy individuals of Chinese lizard gudgeon were captured on 15 October 2025 using benthic seine nets from the mainstream section of the Wuning Xiu River, Jiujiang City, China (114.933480, 29.213640). Fish anesthesia was implemented via tricaine methanesulfonate (MS-222) aqueous solution to minimize physiological stress before sampling. We dissected and collected muscle tissue from a female individual (~16 cm) for high-quality genomic DNA isolation. Twelve types of tissue samples, including 11 (muscle, gill, liver, ovary, brain, skin, intestine, eye, heart, head kidney and bone) from the female individual and one (testis) from a male individual, were rapidly dissected on ice blocks and flash-frozen in liquid nitrogen for total RNA extraction. All animal experimental protocols were reviewed and approved by the Institutional Animal Care and Use Committee of Jiujiang Academy of Agricultural Sciences (No. JJARS-2025-12), complying with the national standards for laboratory animal welfare and fishery organism sampling ethics.

### 2.2. Nucleic Acid Extraction and Quality Control

Muscle tissue sampled from a female Chinese lizard gudgeon was used for high-molecular-weight (HMW) genomic DNA extraction using MagAttract HMW DNA Kit (Qiagen, Hilden, Germany). Three-tier quality screening was implemented: pulsed-field gel electrophoresis (PFGE) confirmed dominant fragments exceeding 50 kb; NanoDrop One spectrophotometry ensured OD_260_/OD_280_ between 1.8 and 2.0 and OD_260_/OD_230_ above 2.1 to rule out polysaccharide and phenolic impurities; and Qubit 4.0 fluorometry precisely measured DNA concentration, and only extracts with concentrations >150 ng μL^−1^ and negligible fragmentation proceeded to long-read library construction.

Total RNA was separately isolated from twelve tissue types via TRIzol reagent (Invitrogen, Carlsbad, CA, USA). RNA integrity was validated on an Agilent 2100 Bioanalyzer, retaining specimens with RIN ≥ 7.0 and 28S/18S rRNA ratio ≥ 1.6. The twelve extracted RNA samples were separated into two groups. Equivalent RNA amounts of each sample in each group were mixed to prepare two pooled samples subjected to mRNA library preparation.

### 2.3. Library Construction and Sequencing

Qualified HMW gDNA was fragmented to 300–350 bp via focused ultrasonication, followed by terminal repair, A-tailing, and paired-end adapter ligation. Fragments with the target insert size were screened through magnetic bead size selection, amplified by limited-cycle PCR, and purified to construct a PE150 short-read library. The library was sequenced on the DNBSEQ-T7 platform (MGI, Shenzhen, China) for genome *k*-mer spectrum estimation, heterozygosity evaluation, and subsequent genome polishing correction.

Intact HMW gDNA was fragmented using a Megaruptor ultra-shear instrument to obtain 15–23 kb target fragments. SMRTbell linear hairpin adapters were ligated after end repair and damage end removal, followed by exonuclease digestion to eliminate incomplete template fragments. Purified SMRTbell templates were annealed with sequencing primers and bound with DNA polymerase to construct the HiFi library, which was sequenced on the PacBio Revio platform (Pacific Biosciences, Menlo Park, CA, USA) to generate high-precision circular consensus long reads (CCSs).

Fresh frozen muscle tissue cells from the same female Chinese lizard gudgeon were cross-linked with formaldehyde to fix chromatin spatial interaction structures, then lysed to extract intact nuclei. Restriction enzyme DpnII was used for chromatin digestion; biotin-labeled nucleotides were filled at sticky ends, followed by intramolecular cyclization ligation under a diluted system to avoid intermolecular cross-linking noise. After cross-link reversal and DNA purification, biotin-enriched interaction fragments were captured by streptavidin magnetic beads, processed for terminal repair and library construction, and sequenced with the PE150 strategy on the DNBSEQ-T7 platform (MGI, Shenzhen, China) to provide chromatin contact information for chromosome-level scaffolding.

Poly-T magnetic beads were used to enrich mRNA molecules with polyA tails from pooled RNA samples. mRNA was fragmented under metal ion thermal conditions. Random hexamer primers were applied for first-strand cDNA synthesis, followed by second-strand cDNA synthesis with a dUTP labeling strategy to retain strand-specific information. End repair, adapter ligation, and fragment screening were conducted, and U-containing second-strand cDNA was degraded during PCR amplification to build a strand-specific RNA-seq library, which was sequenced on the NovaSeq 6000 platform (Illumina, San Diego, CA, USA).

### 2.4. De Novo Genome Assembly and Quality Assessment

Whole-genome clean short reads were processed to remove low-quality bases and adapter sequences. A 21-mer frequency distribution curve was constructed via KMC v3.2.1 [13], and genome size, heterozygosity ratio, and repetitive sequence proportion were estimated using GenomeScope v2.0 [14], providing parameter reference for subsequent long-read primary assembly.

Filtered PacBio HiFi CCS reads were input into Hifiasm v0.25 (-n-hap 2) [15] for primary haplotype-resolved contig construction. Dual haplotype contig sets generated were distinguished, and redundant allelic contigs were eliminated using purge_haplotigs [16] with default parameters to remove haplotype duplication noise and obtain a non-redundant primary contig set. The contig set was then aligned against the NCBI NT nucleotide database using BLASTn (version 2.13.0+, parameters: -evalue 0.00001 -max_hsps 1). Sequences originating from organelles and exogenous contaminants were identified and filtered out according to the alignment results, ultimately generating a high-quality decontaminated preliminary assembly.

Valid Hi-C interaction paired reads were screened by mapping reads to the corrected contig assembly using BWA-MEM (0.7.17-r1188) [17]; invalid self-ligation fragments and background noise pairs were filtered out according to mapping position characteristics. The 3D-DNA pipeline (-r 0) [18] was adopted to cluster, order, and orient contigs based on the normalized chromatin interaction frequency matrix. Juicebox (v1.11.08) [19] was used for manual visual correction of misassembled contig blocks to eliminate abnormal interaction signals and redundant contigs. Unanchored small contigs were classified as unplaced scaffolds for separate statistics.

The BUSCO v6.0 [20] pipeline with the actinopterygii_odb12 orthologous gene dataset was used to assess gene-level genome completeness. Whole-genome short reads and PacBio HiFi long reads were separately mapped to the assembled genome using BWA-MEM [17] and Minimap2 [21] to count mapping rate, coverage depth, and uniform coverage distribution, respectively. Contig N50, scaffold N50, total assembly length, and other continuity indicators were calculated as core assembly metrics.

### 2.5. Genome Annotation

RepeatModeler [22] and LTR_FINDER_parallel [23] were used to construct a species-specific transposable element (TE) library via ab initio prediction; RepBase (http://www.girinst.org/repbase (accessed on 25 May 2026)) was introduced for homologous TE identification. The two repeat libraries were merged and de-redundant, and then RepeatMasker v4.2.1 [24] was run across the whole chromosome sequence to label all transposon regions. Tandem repetitive sequences including microsatellites were separately annotated by TRF v4.10 [25], and the proportion, length distribution, and classification statistics of all repeat elements were summarized.

Gene structural annotation was first implemented to identify the exact genomic location and structural characteristics, followed by functional annotation to characterize the biological functions of gene products and their involved metabolic pathways. For accurate structural prediction of protein-coding genes, three lines of evidence were integrated for gene model prediction, including homology-based prediction, transcriptome-supported prediction and ab initio prediction.

Homology-based gene prediction was conducted using five closely related species (including *Gobio gobio*, *Pseudorasbora parva*, *Danio rerio*, *Cyprinus carpio*, *Puntigrus tetrazona*) as references. miniprot v0.11 [26] was first employed for protein-to-genome alignment to identify conserved gene structures across all five reference species. Liftoff v1.6.3 [27] was subsequently applied to *G. gobio* and *P. parva* for reference-guided annotation mapping. De novo gene prediction was performed utilizing multiple ab initio prediction tools, including AUGUSTUS [28] for universal gene prediction and Genscan [29] optimized for animal genome annotation. Transcriptome-based evidence was incorporated to refine gene models: RNA-seq data were mapped to the assembled genome via HISAT2 [30], followed by transcript assembly using StringTie [31]. TransDecoder (https://github.com/TransDecoder/TransDecoder (accessed on 25 May 2026)) was further applied to predict precise coding regions from the assembled short-read transcriptomes. BUSCO v6.0 [20] was used to evaluate and optimize gene prediction results, which adopted the self-training mode of AUGUSTUS [28] and conserved orthologous gene databases corresponding to the taxonomic classification of the studied species to improve the accuracy of predicted gene structures. All gene sets derived from homology-based, de novo, and transcriptome-based predictions were integrated and filtered by MAKER2 [32] to generate a non-redundant and refined preliminary gene set. Finally, the in-house developed HiFAP (Hierarchical Integrative Framework for Annotation Prioritization) [33] was utilized to generate a high-confidence gene set through systematic evidence reconciliation, and a high-quality final gene repertoire was obtained for subsequent functional annotation. For functional annotation, predicted protein sequences were aligned against NR, SwissProt, TrEMBL, KOG, AnimalTFDB, GO, KEGG, Pfam and InterPro databases. GO terms, enzyme codes, conserved domains, and metabolic pathway information were assigned to each gene.

tRNAs were identified using tRNAscan-SE v2.0.12 [34], which detects the canonical cloverleaf secondary structure and conserved sequence motifs via covariance model scoring. rRNAs were annotated using two complementary tools: Barrnap v0.9 [35] for rapid model-based prediction and Infernal v1.1.5 [36] for homology-based detection against the Rfam database v15.0 [37]; overlapping loci were resolved by retaining the annotation with the lowest e-value. miRNAs and snRNAs were annotated using Infernal v1.1.5 with Rfam v15.0. All ncRNA loci were mapped to chromosomes and counted for copy number, average length, and genome occupancy.

### 2.6. Comparative Evolutionary Genomics Analysis

Genome protein datasets of 11 species, including 10 representative cypriniform species covering *Saurogobio dabryi*, *Gobio gobio*, *Pseudorasbora parva*, *Ctenopharyngodon idella*, *Megalobrama amblycephala*, *Danio rerio*, *Onychostoma macrolepis*, *Puntigrus tetrazona*, *Labeo rohita*, *Cirrhinus molitorella*, and an outgroup species *Oreochromis niloticus*, were collected from NCBI datasets. All-versus-all BLASTP was conducted for protein sequence similarity comparison, then OrthoFinder v2.5.5 [38] was used to cluster orthologous and paralogous gene families, and single-copy orthologous gene sets were extracted for subsequent phylogeny construction. Single-copy gene families were identified and filtered to exclude families encoding proteins shorter than 100 amino acids. For each retained single-copy gene family, protein sequences were aligned using MAFFT v7.525 [39], and the resulting alignments were subsequently reverse-translated into corresponding coding sequence (CDS) alignments.

Three distinct concatenation strategies were employed to construct the super-alignment matrix for comparative genomic analysis: (i) Only-linking method: alignments of all single-copy genes were directly concatenated without further filtering; (ii) Gblocks_method1: all single-gene alignments were first concatenated, and conserved blocks were subsequently extracted using Gblocks v0.91b [40]; and (iii) Gblocks_method2: conserved blocks were extracted individually for each gene alignment using Gblocks [40], followed by concatenation of the filtered alignments. Gene families with a retained sequence ratio below 0.3 relative to the original alignment were excluded from this strategy. For each of the three super-alignment matrices, three site-specific datasets were generated: (a) all sites (full unfiltered matrix), (b) phase 1 sites (first-codon positions), and (c) fourfold degenerate (4D) sites, yielding a total of nine datasets. Maximum likelihood (ML) phylogenetic trees were inferred independently for each of the nine datasets using RAxML [41]. The final species phylogeny was determined based on topological congruence among the nine resulting trees and consistency with well-established species relationships.

Species divergence times were estimated using the MCMCTree program implemented in the PAML v4.9j package [42], based on fourfold degenerate (4D) sites extracted from single-copy gene families. Four calibration points from TimeTree (http://www.timetree.org) were applied to constrain molecular clock dating: the divergence of *Oreochromis niloticus* and *Puntigrus tetrazona* was calibrated at 224.0 Mya (180.0–251.5 Mya); *Danio rerio* and *Megalobrama amblycephala* at 44.9 Mya (41.4–51.0 Mya); *Pseudorasbora parva* and *Gobio gobio* at 23.32 Mya (8.87–34.94 Mya); and *Megalobrama amblycephala* and *Ctenopharyngodon idella* at 21.74 Mya (9.10–29.82 Mya).

Gene family expansion and contraction analysis was conducted using the CAFE v5.0 pipeline [43], based on the time-calibrated phylogenetic tree and orthologous gene clusters. Briefly, all-versus-all BLASTP alignment was first performed across protein sequences of all selected species, and orthologous gene families were clustered via OrthoFinder v2.5.5 [38] with default parameters. Gene families present in fewer than three species or with extreme copy number outliers were filtered out prior to analysis to reduce estimation bias. The global gene birth–death rate (λ) was estimated by maximum likelihood, and the numbers of expanded and contracted gene families on each phylogenetic branch were calculated. Gene families with a *p*-value < 0.05 were defined as significantly expanded or contracted.

Single-copy orthologous gene alignments were converted to codon sequences; the branch-site model of PAML v4.9j [42] was applied to calculate ω (dN/dS) ratios of the foreground branch (genus *Saurogobio*) and background cyprinid branches, screening positively selected genes (PSGs) with significant statistical support. To further interpret the biological implications of lineage-specific gene family dynamics, genes were categorized into functional groups and biological pathways following the official classification criteria of Gene Ontology (GO) and the KEGG database, respectively. Functional enrichment analyses for both GO terms and KEGG pathways were implemented using the R program v4.5.0 package clusterProfiler [44]. Raw *p*-values obtained from enrichment tests were subjected to FDR correction to generate adjusted *p*-values (p.adjust) and q-values for subsequent significance assessment.

Whole-genome synteny analysis was performed using the WGDI toolkit v0.5.6 [45]. Briefly, all-vs-all BLASTP alignment was conducted between the protein sequences of *Saurogobio dabryi* and *Gobio gobio* with an E-value cutoff of 1 × 10^−5^, and gene coordinate information as well as chromosome length data were extracted as input files. Collinear genomic blocks were subsequently identified and statistically summarized via WGDI, and the genome-wide syntenic relationships were visualized using the JCVI v1.1.22 [46].

## 3. Results

### 3.1. Genome Size and Sequencing Data Summary

We first estimated the genome characteristics of *S. dabryi* based on the 21-mer frequency distribution of 155.45 Gb whole-genome clean short reads. The *k*-mer coverage profile displayed two distinct peaks corresponding to heterozygous and homozygous genomic regions, consistent with a diploid genome model (Figure 1B). GenomeScope analysis predicted an estimated genome size of 1.047 Gb, with a repeat sequence ratio of 40.90% and a heterozygosity rate of 0.912% (Figure 1B). The low error rate (0.099%) and high model fit validated the reliability of genomic parameter estimation, indicating moderate heterozygosity and abundant repetitive elements in the *S. dabryi* genome.

For whole-genome sequencing, we generated a total of 61.06 Gb of high-precision PacBio HiFi clean reads with an average fragment length of 17.60 kb (~56× genome coverage), and 155.45 Gb of clean short-read data (~142× genome coverage). Global quality profiling showed that all sequencing data possessed a Q30 base accuracy exceeding 98.09%, along with uniform GC distribution across the entire dataset, demonstrating stable sequencing performance and the absence of systematic bias and exogenous contamination. For chromosome scaffolding, we additionally generated 165.98 Gb of clean Hi-C sequencing data (~152× genome coverage) and 12.63 Gb of multi-tissue RNA-seq data to support gene annotation (Table 1).

### 3.2. Chromosome-Level Genome Assembly and Quality Validation

De novo genome assembly was performed using 3.46 million PacBio HiFi reads to generate CCS reads. The initial contig assembly reached a total length of 1.11 Gb, with a contig N50 of 40.98 Mb and a maximum contig length of 79.60 Mb, indicating superior assembly continuity (Table S1). After eliminating redundant allelic contigs and contamination, a total of 158 high-quality contigs with total length reaching 1,110,283,590 bp were obtained as the de novo genome assembly of *S. dabryi* (Table S1). Remapping short reads and PacBio HiFi long reads to the assembled genome resulted in a 99.58% mapping rate and 137.07× average depth, covering 99.84% of the genome for the short reads, and a 99.96% mapping rate, 53.46× depth, and full 100% genome coverage for the long reads, indicating high accuracy of the primary assembly.

**Figure 2:**
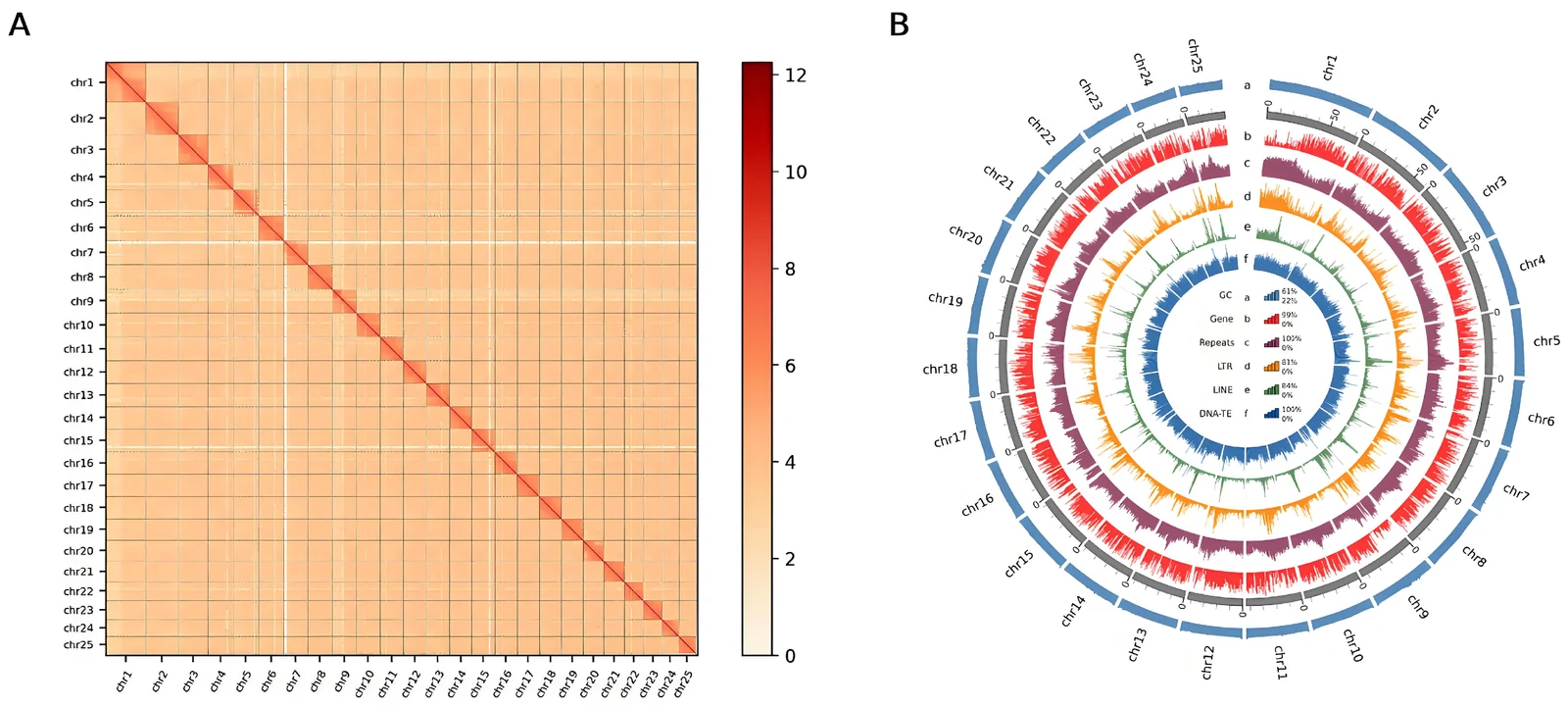
Hi-C contact heatmap and circular genomic landscape of chromosome-level assembly of the Chinese lizard gudgeon (*Saurogobio dabryi*). (**A**) Hi-C interaction heatmap of the 25 chromosome scaffolds. The horizontal and vertical axes correspond to the ordered chromosomes (chr1–chr25). Color gradient from pale yellow to deep red indicates the intensity of chromatin contact frequency (color scale bar on the right). Diagonal blocks represent strong intra-chromosomal interactions, while sparse off-diagonal signals reflect weak inter-chromosomal contacts, verifying the high-quality chromosome-scale genome assembly without severe misjoins. (**B**) Circular multi-track genomic overview of 25 chromosomes of *S. dabryi*. Tracks from outer to inner: (a) GC content distribution; (b) protein-coding gene density; (c) total repetitive element coverage; (d) LTR transposon density; (e) LINE transposon density; (f) DNA-TE transposon density. The embedded bar chart in the circle center shows the genome-wide average proportion of each genomic feature corresponding to tracks a–f.

To scaffold the draft contigs to chromosomal scale, Hi-C interaction reads were mapped to the polished contig set, and contigs were clustered, ordered, and oriented, followed by manual curation. The Hi-C interaction heatmap displayed distinct diagonal enrichment of intra-chromosomal chromatin interactions and minimal inter-chromosomal noise, strongly supporting the high accuracy and structural integrity of the chromosomal assembly (Figure 2A). In total, 66 contigs were successfully anchored and oriented onto 25 chromosomes, with the remaining 80 contigs retained as unplaced scaffolds (Figure 2B and Figure S1, Table 2). The final whole-genome assembly comprised 146 contigs and 105 scaffolds, with a total length of 1,095,455,107 bp. The sequences anchored to chromosomes accounted for 99.55% of the total assembled genome, consistent with the documented karyotypic characteristics of *S. dabryi* [47]. The final chromosome-level genome assembly yielded a scaffold N50 of 43.15 Mb and a contig N50 of 40.38 Mb (Table 2). BUSCO assessment using the actinopterygii_odb12 dataset achieved a complete BUSCO rate of 98.39% (Table S2).

### 3.3. Genome Annotation Features

Repetitive sequence annotation was performed via combined de novo and homology-based strategies. Total repetitive sequences (659.55 Mb) occupied 60.21% of the entire assembled genome (Table S3). We annotated 16.09 Mb satellite repeat sequence across the genome (Table S4). Class II DNA transposons represented the most abundant TE superfamily, accounting for 34.37% of the genome (Table S5, Figure 2B), whereas Class I retrotransposons were mainly dominated by Gypsy-type LTR elements (122.82 Mb, 11.21% of the genome), accompanied by low-abundance LINE (63.84 Mb, 5.83% of the genome) and SINE fragments (7.56 Mb, 0.69% of the genome).

Protein-coding gene prediction was conducted by integrating three independent annotation strategies to ensure the accuracy and integrity of gene models. In total, 26,036 protein-coding genes were reliably annotated in the *S. dabryi* genome (Table S6), with an average gene length of 21.75 kb, average CDS length of 1.74 kb, an average of 9.99 exons per gene, and an average exon length of 264.20 bp (Figure S2). BUSCO assessment on the final gene set reached 97.77% completeness using the actinopterygii_odb12 dataset, verifying high annotation integrity (Table S7). After alignments to multiple databases, 25,898 protein-coding genes (99.47% of all predicted genes) obtained definite functional annotations, with only a tiny fraction of genes remaining uncharacterized due to lineage-specific divergence.

We systematically annotated multiple categories of non-coding RNAs (ncRNAs) across the *S. dabryi* genome (Table S8). rRNA and tRNA constituted the dominant ncRNA components, occupying 0.263% and 0.259% of the whole genome, respectively. A total of 20,545 rRNA copies were detected, consisting of 5S rRNA (20,283 copies), 5.8S rRNA (60 copies), 18S rRNA (90 copies) and 28S rRNA (112 copies). We predicted 37,382 tRNA loci with an average length of 76 bp. Other minor ncRNA groups included 1,320 miRNAs (0.008%), 2,601 snRNAs (0.034%), and merely 11 scaRNA copies (0.0002%).

### 3.4. Comparative Genomic Analysis

**Figure 3:**
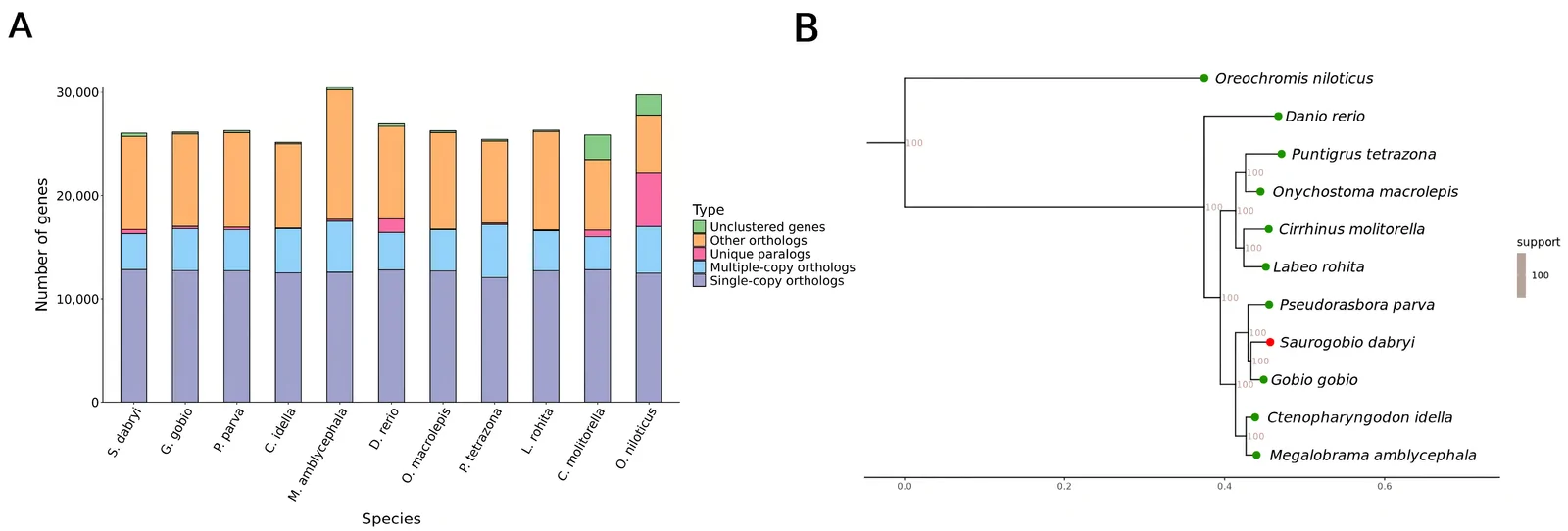
Orthologous gene classification and phylogenetic relationships of examined cypriniform species. (**A**) Stacked bar chart showing the categorization and total counts of gene repertoires across 11 fish species. Each stacked segment represents one gene category: light purple = single-copy orthologs; light blue = multiple-copy orthologs; magenta = unique paralogs; orange = other orthologs; light green = unclustered genes. The vertical axis denotes total gene number, and the horizontal axis lists the sampled species. (**B**) Maximum-likelihood phylogenetic tree constructed using single-copy orthologous genes from all taxa. All internal branches received maximal support values of 100 (bootstrap = 100). The focal species *Saurogobio dabryi* is highlighted with a red terminal dot, while green dots mark all other species. The horizontal scale bar represents genetic distance, and the grey gradient bar on the right illustrates the range of nodal support values.

Orthologous gene family clustering was performed across 11 species, including 10 representative Cypriniformes species and one Cichliformes species (*Oreochromis niloticus*) as the outgroup. A total of 14,107 common gene families were identified, among which 10,510 single-copy orthologous genes were shared by all species (Figure 3A, Table S9). A maximum-likelihood phylogenetic tree was constructed based on the 10,510 single-copy orthologous genes, with 100 bootstrap replicates supporting all major nodes. The focal species *S. dabryi* was stably nested within the monophyletic clade of Gobioninae and formed sister species with *Gobio gobio* (Figure 3B). These two taxa jointly constituted a *Gobio*–*Saurogobio* lineage, which further clustered with *Pseudorasbora parva* (Figure 3B). The recovered topology confirmed that *Saurogobio* shares the closest phylogenetic affinity with *Gobio*, and both genera belong to the Gobioninae lineage, which is fully consistent with previous morphological taxonomy and molecular phylogenetic conclusions [12]. All nodes of closely related taxa achieved maximal support without topological conflicts, providing a solid topological basis for subsequent divergence time calibration.

The divergence time separating *Pseudorasbora parva* from the clade containing *Saurogobio* and *Gobio* was estimated at 32.0 Mya (95% HPD: 27.5–37.6 Mya, Figure S3). The interspecific divergence between *S. dabryi* and *Gobio gobio* was dated to 12.6 Mya (95% HPD: 11.0–14.3 Mya, Figure S3), corresponding to the Middle Miocene. This geological interval represents a critical epoch for speciation of East Asian freshwater fishes, coinciding with episodic uplift of the Qinghai–Tibet Plateau and frequent fragmentation and reorganization of drainage systems across the middle and lower reaches of the Yangtze River [48].

**Figure 4:**
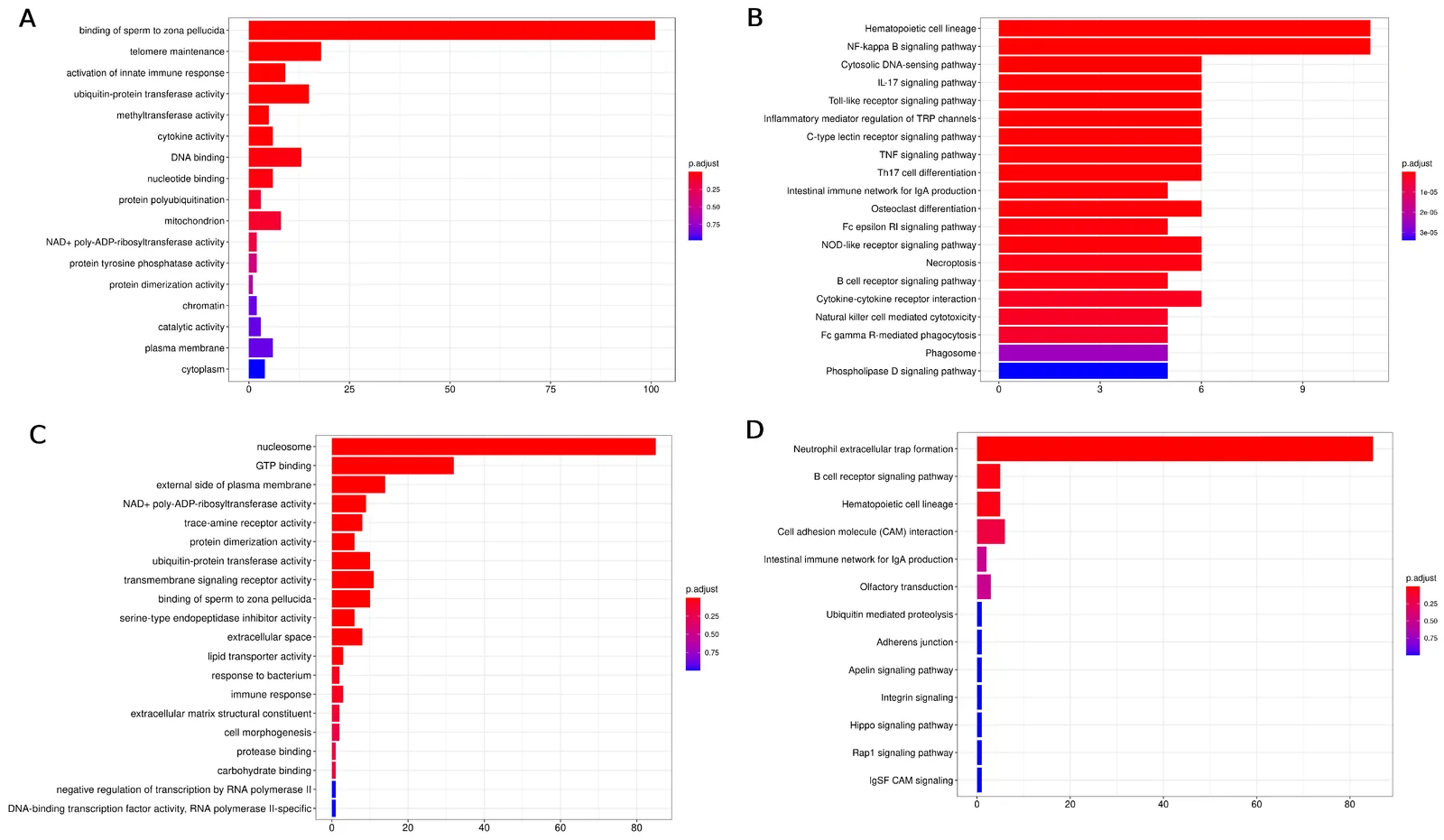
Functional enrichment analyses of significantly expanded and contracted gene families in *Saurogobio dabryi*. (**A**) Top enriched GO functional categories for significantly expanded gene families in *S. dabryi*; horizontal bar length represents the number of enriched genes, and the color gradient from red to blue corresponds to adjusted *p*-value. (**B**) Top enriched Kyoto Encyclopedia of Genes and Genomes (KEGG) pathways for significantly expanded gene families in *S. dabryi*, with bar width indicating enriched gene count and color shading reflecting adjusted significance. (**C**) Top enriched GO functional categories for significantly contracted gene families in *S. dabryi*. (**D**) Top enriched KEGG pathways for significantly contracted gene families in *S. dabryi*. The vertical axes list functional GO terms or KEGG pathway names; the horizontal axes show the count of enriched genes; color bars on the right of each panel denote the gradient of adjusted *p*-values.

To decipher the genomic evolutionary basis of *S. dabryi*, we performed comparative genomic analysis of orthologous gene families across representative Cypriniformes species, and quantified lineage-specific gene gain and loss events based on the time-calibrated phylogeny established above. The branch leading to *S. dabryi* was designated as the focal lineage for screening significantly expanded and contracted gene families (*p* < 0.05). A total of 171 expanded gene families and 590 contracted gene families were detected on the *S. dabryi* branch (Figure S4). Compared with pelagic cyprinids such as *Ctenopharyngodon idella* and *Megalobrama amblycephala*, the *S. dabryi* lineage exhibited a markedly higher number of expanded gene families, whereas its closely related congener *Gobio gobio* displayed a milder expansion signal and more extensive gene family contraction (Figure S4). Such intergeneric differentiation indicated that lineage-specific gene duplication and gene loss have reshaped the genomic repertoire of *S. dabryi* after its Middle Miocene split from *G. gobio*. Functional enrichment analysis showed that significantly expanded gene families were mainly enriched in pathways related to binding of sperm to zona pellucida, telomere maintenance and hematopoietic cell lineage (Figure 4A,B), and significantly contracted gene families were mainly enriched in nucleosome, GTP binding and neutrophil extracellular trap formation (Figure 4C,D).

We identified 292 positively selected genes (PSGs) in the *S. dabryi* lineage. The PSGs in *S. dabryi* were significantly enriched in GO functional categories such as plasma membrane, guanyl-nucleotide exchange factor activity and actin binding (Figure S5A) and KEGG pathways such as spliceosome, parathyroid hormone synthesis, secretion and action, and endocytosis (Figure S5B).

**Figure 5:**
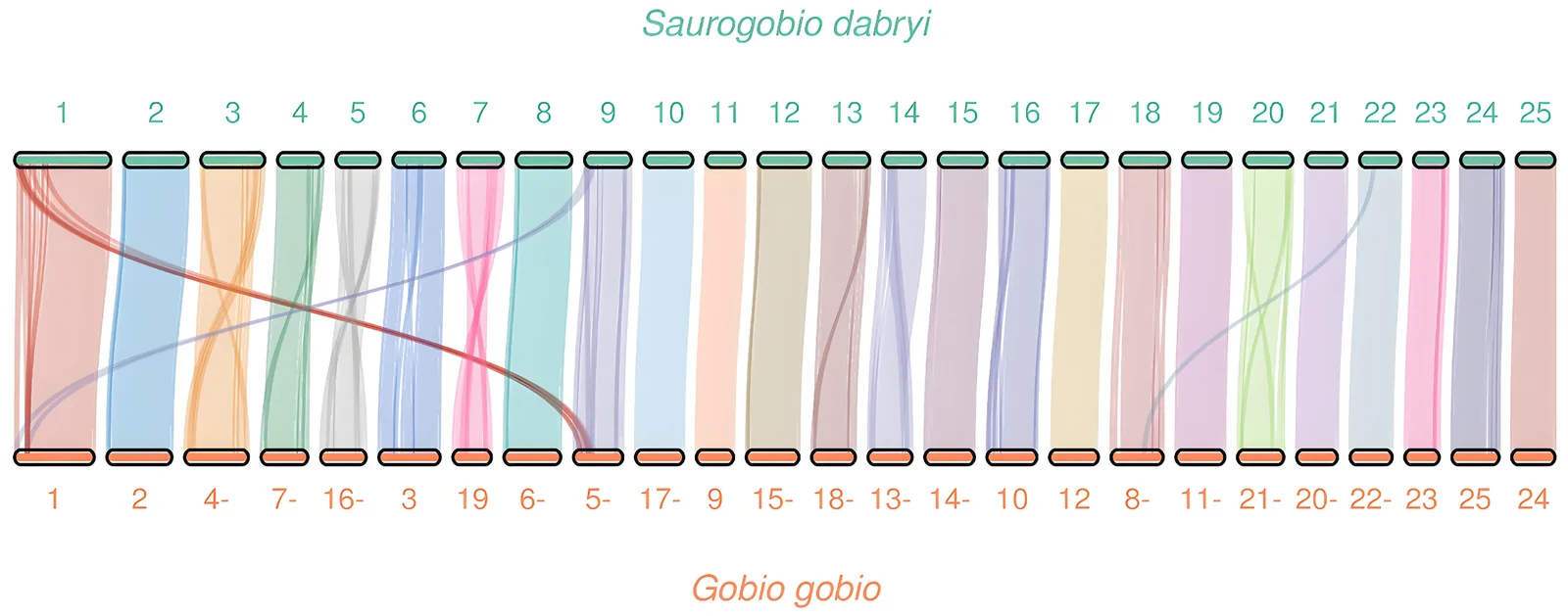
Whole-genome synteny chord diagram between *Saurogobio dabryi* and its sister species *Gobio gobio*. The upper row of vertical-colored bars corresponds to the 25 chromosomes of *S. dabryi*, labeled numerically from 1 to 25; the lower row of vertical bars represents orthologous chromosomes of *G. gobio*, with chromosome identifiers annotated beneath each bar. Colored interconnecting ribbons indicate conserved syntenic orthologous genomic segments shared between the two species, where ribbon thickness corresponds to the length of collinear blocks. Hyphen after chromosome number means the reverse of the chromosome. Intersecting ribbons denote inter-chromosomal translocations and large-scale chromosomal rearrangement events accumulated after the lineage divergence of *S. dabryi* and *G. gobio*.

We performed whole-chromosome synteny analysis between *Saurogobio dabryi* and *Gobio gobio* (Figure 5). Broad conserved synteny was observed across most chromosomes between the two closely related Gobioninae species. Multiple chromosomes retain large contiguous orthologous blocks, yet inter-chromosomal syntenic ribbons are widely detected, revealing extensive inter-chromosomal translocations accumulated after their Middle Miocene divergence (12.6 Mya). For instance, chromosomes 10, 11, 17, 21 and 25 of *S. dabryi* displayed unbroken single-block synteny with their respective orthologous counterparts in *G. gobio*, with no inter-chromosomal translocation signals detected.

## 4. Discussion

In this study, we present the first chromosome-level reference genome of *S. dabryi*, a representative benthic Gobioninae fish endemic to East Asia. The assembly exhibits high continuity and completeness, and chromosome-level scaffolding, representing one of the highest-quality genome assemblies for Gobioninae species to date.

GO and KEGG enrichment analyses of the 171 significantly expanded gene families revealed two dominant functional themes: immune defense and sperm–egg recognition. The most significantly enriched GO term was “binding of sperm to zona pellucida” (101 genes, p.adjust = 8.91 × 10^−121^), indicating massive expansion of ZP-domain proteins mediating fertilization.

The more striking signal was a coordinated expansion across nearly all compartments of the immune system. At the GO level, “activation of innate immune response” (9 genes, p.adjust = 2.11 × 10^−8^) and “cytokine activity” (6 genes, p.adjust = 6.33 × 10^−3^) were enriched. At the KEGG level, enrichment spanned pattern-recognition receptor pathways (TLR, NOD-like, C-type lectin, cytosolic DNA sensing), pro-inflammatory signaling axes (NF-κB, TNF, IL-17, MAPK), lymphocyte development and function (hematopoietic cell lineage, B-cell receptor, Th17 differentiation, IgA network, NK cytotoxicity), and myeloid effector mechanisms (phagocytosis, phagosome, NET formation), with the strongest signals in hematopoietic cell lineage (11 genes, p.adjust = 1.25 × 10^−17^) and NF-κB signaling (11 genes, p.adjust = 6.23 × 10^−17^). Upstream cascades including calcium and PI3K-Akt signaling were also enriched. This pervasive expansion across both innate and adaptive immunity suggests sustained selection for enhanced immunocompetence rather than isolated pathway duplication.

We hypothesize that this immune gene expansion is associated with the benthic ecology of *S. dabryi*. As a bottom-dwelling fish inhabiting sandy and silty substrates, it likely encounters a higher load of sediment-associated pathogens and microbes than pelagic species, with the IgA intestinal immune network further pointing to mucosal and gut-associated immunity as particular targets of selection. We acknowledge that gene family expansion alone does not confirm functional enhancement; expression profiling and selection pressure analyses on individual paralogs will be needed to validate this hypothesis.

Compared with the 171 expanded gene families, 590 gene families were significantly contracted on the *S. dabryi* lineage, indicating that gene loss has contributed substantially to shaping its genomic repertoire. The contraction of nucleosome-associated genes, primarily histones, may reflect a reduction in histone repeat copy number, although partial assembly collapse in these tandemly repeated regions cannot be excluded. GTP-binding protein contraction suggests streamlining of certain signal transduction pathways, potentially related to relaxed selection on traits less relevant to benthic life. Overall, the predominance of gene family contraction, alongside the targeted expansion of immune and reproductive pathways, underscores a lineage-specific evolutionary trajectory shaped by both gene gain and gene loss following the divergence of *S. dabryi* from its Gobioninae relatives.

Combining the well-supported phylogenetic topology and molecular clock-based divergence times, the present study explicitly clarified the evolutionary position and speciation age of *Saurogobio dabryi*. Topologically, maximal nodal support verified that genus *Saurogobio* is embedded within Gobioninae and forms a sister lineage to *Gobio*. This result resolves intergeneric phylogenetic relationships within Gobioninae and supplements basic phylogenetic data for endemic East Asian benthic gobionids. Temporally, the divergence between *S. dabryi* and its close gobionid relatives took place in the Middle Miocene (12.6 Mya). During this period, drastic restructuring of drainage landscapes across East Asia generated geographic barriers including main river channels, tributaries and lacustrine isolates in the Yangtze River basin [47], which restricted interpopulation gene flow and probably drove independent speciation of *Saurogobio*.

## 5. Conclusions

We successfully constructed the first chromosome-level high-quality reference genome of *S. dabryi* by integrating PacBio HiFi long reads, short sequencing reads, and Hi-C sequencing technology. The final assembly spans 1.09 Gb with a scaffold N50 of 43.15 Mb, and 99.55% of the sequences are anchored to 25 chromosomes. We comprehensively annotated repetitive sequences, protein-coding genes, and non-coding RNAs, and clarified the phylogenetic position of *Saurogobio* within Gobioninae via comparative phylogenomic analysis. This high-quality genome provides a fundamental genetic basis for future studies on benthic fish adaptive evolution, germplasm improvement, and wild population conservation.

## Figures and Tables

**Table 1:** Statistics of the sequencing data generated for Chinese lizard gudgeon.

Libraries	Insert Sizes (bp)	Clean Data (Gb)	Coverage (×)
DNBSEQ-T7	350	155.45	~142
PacBio HiFi	17,600	61.06	~56
Hi-C	350	165.98	~152
RNA-Seq	350	12.63	-

**Table 2:** Summary of genome assembly of the Chinese lizard gudgeon.

Index	Number/Size
Genome size (bp)	1,095,455,107
Number of scaffolds	105
N50 scaffold length (bp)	43,148,182
N90 scaffold length (bp)	35,247,163
Max length (bp)	72,897,996
Number of contigs	146
N50 contigs length (bp)	40,375,940
N90 contigs length (bp)	17,567,085
Max length (bp)	72,671,769
GC content (%)	39.18

## Data Availability

The raw sequence data reported in this paper have been deposited in the Genome Sequence Archive in the National Genomics Data Center, China National Center for Bioinformation/Beijing Institute of Genomics, Chinese Academy of Sciences (GSA: CRA046760), which is publicly accessible at https://ngdc.cncb.ac.cn/gsa. The final chromosome-level assembly, annotation files including CDSs, predicted proteins and repeat annotation were deposited in *figshare* with accession (Genome assembly and annotation of the Chinese lizard gudgeon (*Saurogobio dabryi*). figshare. Dataset. https://doi.org/10.6084/m9.figshare.33091439.v2).

## Supplementary Materials

The following supporting information can be downloaded at: https://www.mdpi.com/article/10.3390/ani16172647/s1, Table S1 Statistics of de novo assembled primary genome based on PacBio HiFi sequencing data; Table S2 BUSCO assessment of anchored genome in the *S. dabryi* genome; Table S3 Statistical Results of Repeats annotation in the *S. dabryi* genome; Table S4 Statistical Results of satellite repeat sequence annotation in the *S. dabryi* genome; Table S5 Length and proportion of transposable element superfamilies in the *S. dabryi* genome; Table S6 Basic Statistical Results of Gene Prediction in the *S. dabryi* genome; Table S7 BUSCO assessment of the *S. dabryi* genome annotation; Table S8. Summary statistics of non-coding RNA annotations in the *S. dabryi* genome; Table S9 Statistics of Gene Family Clustering Results in the *S. dabryi* genome; Figure S1 Chromosomal architecture of the *Saurogobio dabryi* genome assembly; Figure S2 Comparison of gene structure features between *Saurogobio dabryi* and five representative cyprinid species; Figure S3 Time-calibrated phylogenetic tree of 11 cypriniform species; Figure S4 Gene family expansion and contraction across the phylogenetic tree of 11 cypriniform species; Figure S5 Functional enrichment analysis of positively selected genes (PSGs) in *Saurogobio dabryi*.
