# Supplementary material for: Chromosome-Level Genome Assembly and Annotation of the Chinese Lizard Gudgeon (Saurogobio dabryi)
Source: Animals (Basel). 2026 Aug 24;16(17):2647. doi: 10.3390/ani16172647 (PMC13564343; doi:10.3390/ani16172647)
Supplement: Supplementary file 1 [file animals-16-02647-s001.zip › animals-4493907-supplementary.pdf]

Supplementary Tables

Table S1 Statistics of de novo assembled primary genome based on PacBio HiFi sequencing data

	**Contig Length (bp)**	**Contig Number**	**Scaffold Length (bp)**	**Scaffold Number**
N90	17,567,085	26	17,567,085	26
N80	26,773,399	21	26,773,399	21
N70	32,313,701	17	32,313,701	17
N60	38,220,802	14	38,220,802	14
N50	40,984,353	11	40,984,353	11
Number(>=100bp)	-	158	-	158
Number(>=2kb)	-	158	-	158
Max length	79,601,128	-	79,601,128	-
Total length	1,110,283,590	-	1,110,283,590	-

Table S2 BUSCO assessment of anchored genome in the *S. dabryi* genome

**Software**	**BUSCO**	
**Type**	**Proteins**	**Percentage (%)**
Complete BUSCOs	7,091	98.39
Complete Single-Copy BUSCOs	7,054	97.88
Complete Duplicated BUSCOs	37	0.51
Fragmented BUSCOs	16	0.22
Missing BUSCOs	100	1.39
Total BUSCO groups searched	7,207	100.00
Database	actinopterygii_odb12	

Table S3 Statistical Results of Repeats annotation in the *S. dabryi* genome

**Type**	**Repeat Size (bp)**	**% of genome**
Trf	116,600,886	10.64
Repeatmasker	350,070,283	31.96
Proteinmask	105,502,051	9.63
De novo	468,225,608	42.74
Total	659,546,439	60.21

Table S4 Statistical Results of satellite repeat sequence annotation in the *S. dabryi* genome

	**RepBase TEs**		**TE Proteins**		**De novo**		**Combined TEs**	
	**Length (bp)**	**%in Genome**	**Length (bp)**	**%in Genome**	**Length (bp)**	**%in Genome**	**Length (bp)**	**%in Genome**
DNA	236,120,705	21.55	45,612,813	4.16	272,156,310	24.84	376,496,893	34.37
LINE	47,714,046	4.36	23,725,795	2.17	37,273,371	3.40	63,839,615	5.83
SINE	6,054,520	0.55	0	0.00	4,107,896	0.37	7,565,406	0.69
LTR	63,168,559	5.77	36,229,819	3.31	85,815,037	7.83	122,817,173	11.21
Satellite	14,452,402	1.32	0	0.00	2,921,845	0.27	16,098,140	1.47
Simple_repeat	0	0.00	0	0.00	0	0.00	0	0.00
Other	124,420	0.01	0	0.00	119,868	0.01	232,792	0.02
Unknown	5,046,170	0.46	165	0.00	74,417,428	6.79	78,332,812	7.15
Total	350,070,283	31.96	105,502,051	9.63	468,225,608	42.74	623,784,839	56.94

Table S5 Length and proportion of transposable element superfamilies in the *S. dabryi* genome

**Supertype**	**Length (bp)**	**% Genome**
DNA/TcMar	83,962,140	7.66
DNA/hAT	83,799,342	7.65
Unknown/Unknown	78,331,754	7.15
DNA/DNA	44,120,031	4.03
LTR/Gypsy	38,888,038	3.55
DNA/Kolobok	38,644,182	3.53
DNA/CMC	37,660,793	3.44
LTR/DIRS	36,546,662	3.34
LINE/L2	28,472,393	2.60
LTR/unknown	26,014,708	2.37
DNA/PIF	25,725,673	2.35
DNA/Crypton	18,134,807	1.66
DNA/Helitron	17,881,300	1.63
Satellite/Satellite	16,046,372	1.46
LINE/Penelope	12,058,480	1.10
LINE/L1	11,668,982	1.07
DNA/IS3EU	9,812,224	0.90
LTR/ERV1	9,451,155	0.86
LINE/Rex-Babar	8,388,491	0.77
DNA/Merlin	7,180,882	0.66
DNA/PiggyBac	6,802,433	0.62
LTR/LTR	6,481,229	0.59
DNA/Sola	5,593,894	0.54
DNA/MULE	5,813,701	0.53
DNA/Novosib	4,822,105	0.44
DNA/Zisupton	4,657,736	0.43
DNA/P	4,597,952	0.42
DNA/Maverick	4,499,095	0.41
LTR/Pao	4,435,670	0.40
DNA/Ginger	3,768,470	0.34

Table S6 Basic Statistical Results of Gene Prediction in the *S. dabryi* genome

	**Gene set**	**Protein coding gene number**	**Average gene length (bp)**	**Average CDS length (bp)**	**Average exon per gene**	**Average exon length (bp)**	**Average intron length (bp)**
De novo	Genscan	27,962	24,368	1,639	8.34	196.51	3,096
	AUGUSTUS	25,400	13,877	1,380	7.54	182.88	1,910
Homolog (miniprot)	*Pseudorasbora parva*	60,509	24,346	1,953	10.72	182.16	2,303
	Gobio gobio	53,679	21,020	1,767	10.03	176.20	2,132
	Danio rerio	103,020	32,004	2,304	12.53	183.94	2,577
	Puntigrus tetrazona	52,613	27,996	2,057	12.04	170.83	2,349
	Cyprinus carpio	88,254	27,084	1,958	11.12	176.05	2,482
Liftoff	*Gobio gobio*	24,228	18,618	1,575	8.68	181.39	2,219
	Pseudorasbora parva	24,549	18,547	1,531	8.24	185.78	2,349
Trans ORF	RNA-seq	19,234	27,320	1,907	11.56	293.38	2,266
	BUSCO	7,128	17,672	1,752	11.04	158.66	1,585
	MAKER	26,919	23,212	1,632	9.96	282.93	2,275
	HiFAP	26,036	21,752	1,742	9.99	264.20	2,126

Table S7 BUSCO assessment of the *S. dabryi* genome annotation

**Software**	**BUSCO**	
**Type**	**Proteins**	**Percentage (%)**
Complete BUSCOs	7,046	97.77
Complete Single-Copy BUSCOs	7,006	97.21
Complete Duplicated BUSCOs	40	0.56
Fragmented BUSCOs	28	0.39
Missing BUSCOs	133	1.85
Total BUSCO groups searched	7,207	100.00
Database	actinopterygii_odb12	

Table S8. Summary statistics of non-coding RNA annotations in the *S. dabryi* genome

Type		Copy	Average length (bp)	Total length (bp)	Percentage of genome (%)
miRNA		1,320	72	94,688	0.008644
tRNA		37,382	76	2,846,545	0.259850
rRNA	rRNA	20,545	141	2,888,809	0.263709
	18S	90	1,348	121,346	0.011077
	28S	112	3,335	373,482	0.034094
	5.8S	60	152	9,098	0.000831
	5S	20,283	118	2,384,883	0.217707
snRNA	snRNA	2,601	146	380,704	0.034753
	CD-box	425	173	73,688	0.006727
	HACA-box	76	150	11,420	0.001042
	splicing	2,089	140	293,307	0.026775
	scaRNA	11	208	2,289	0.000209

Table S9 Statistics of Gene Family Clustering Results in the *S. dabryi* genome

**Species**	**Genes number**	**Unclustered genes**	**Genes in families**	**Family number**	**Unique families**	**Unique families genes**	**Common families**	**Common families genes**	**Single copy genes**	**Average genes per family**
S. dabryi	26,036	307	25,729	20,325	65	389	14,107	16,325	10,510	1.266
G. gobio	26,127	160	25,967	20,058	34	216	14,107	16,807	10,510	1.295
P. parva	26,273	186	26,087	20,313	51	252	14,107	16,696	10,510	1.284
C. idella	25,150	131	25,019	19,885	12	49	14,107	16,811	10,510	1.258
M. amblycephala	30,472	218	30,254	20,775	49	178	14,107	17,528	10,510	1.456
D. rerio	26,924	241	26,683	20,075	114	1315	14,107	16,434	10,510	1.329
O. macrolepis	26,254	165	26,089	20,375	27	70	14,107	16,689	10,510	1.280
P.tetrazona	25,449	163	25,286	20,045	46	130	14,107	17,208	10,510	1.261
L. rohita	26,315	138	26,177	20,490	33	85	14,107	16,591	10,510	1.278
C. molitorella	25,865	2,377	23,488	19,382	105	635	14,107	16,022	10,510	1.212
O. niloticus	29,762	1,992	27,770	17,778	527	5,160	14,107	17,010	10,510	1.562

Supplementary Figures

Figure S1 Chromosomal architecture of the *Saurogobio dabryi* genome assembly.

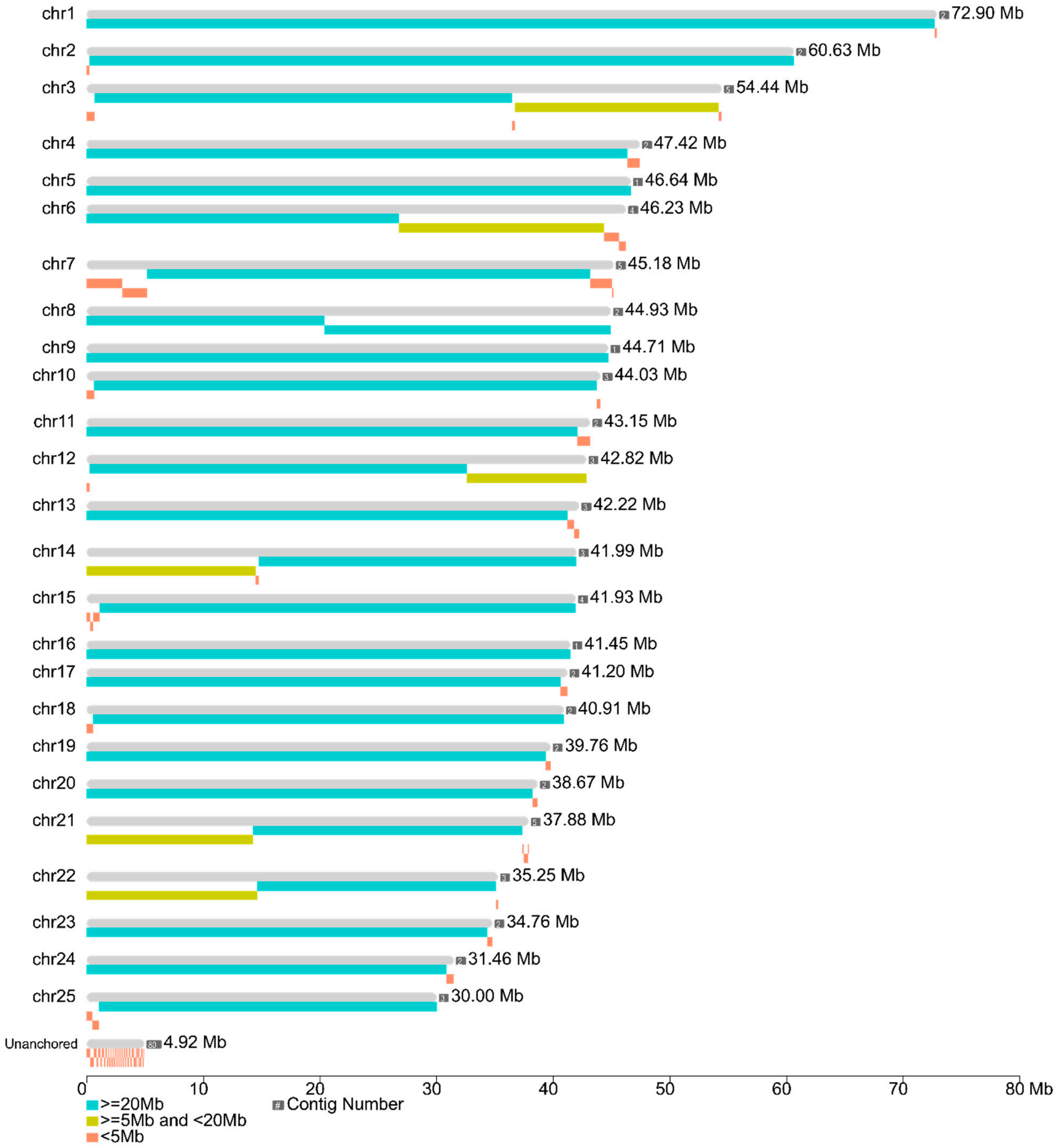

Each horizontal bar represents one of the 25 chromosomes, with colored segments indicating contigs grouped by size (cyan: ≥20 Mb; yellow: 5–20 Mb; orange: <5 Mb). Grey regions denote gaps between adjacent contigs. Chromosome lengths (Mb) are labeled on the right, and unanchored scaffolds are shown at the bottom.

Figure S2 Comparison of gene structure features between *Saurogobio dabryi* and five representative cyprinid species

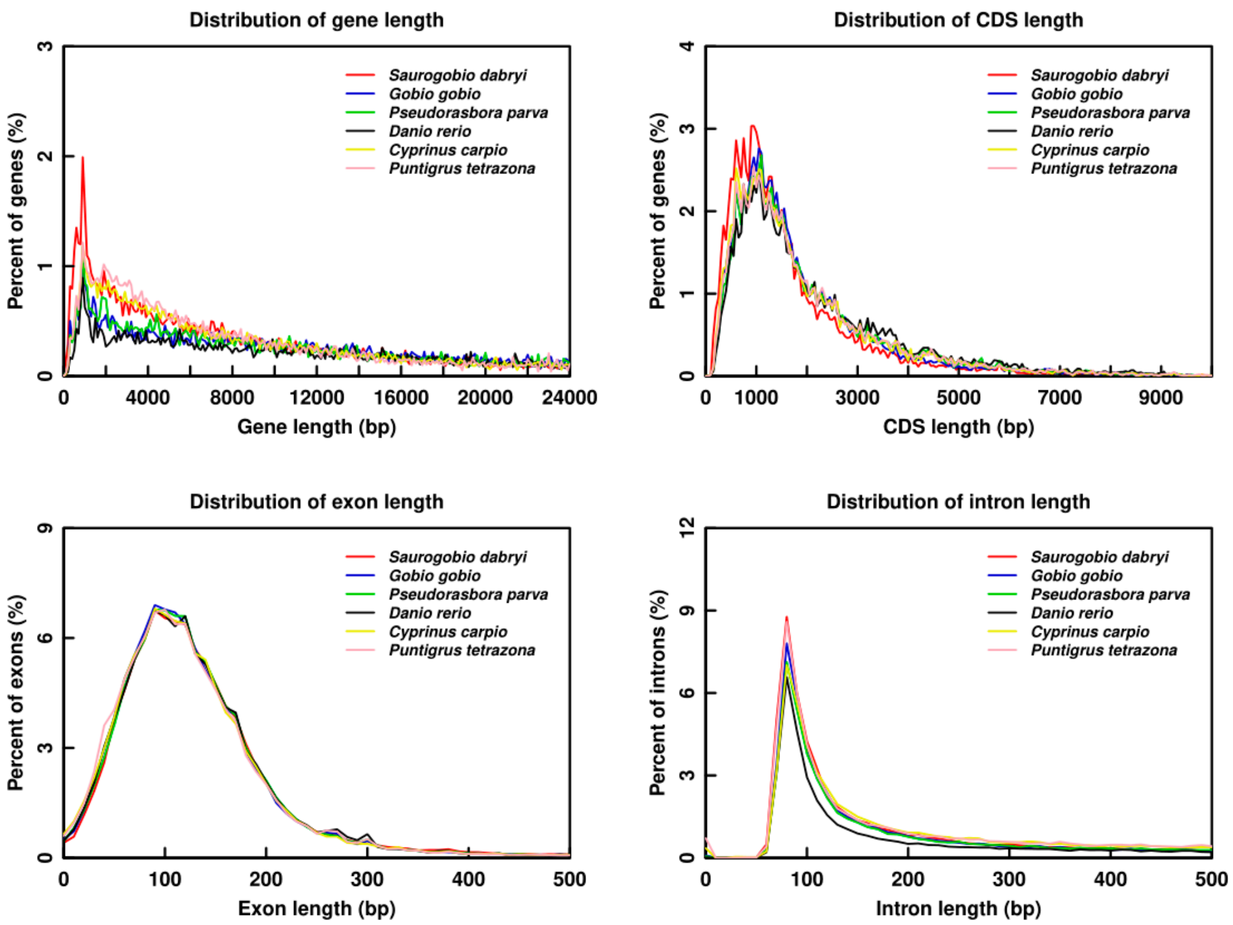

(A) Distribution of gene length; (B) distribution of CDS length; (C) distribution of exon length; (D) distribution of intron length. Each curve represents one species, with the percentage of genes/exons/introns plotted against sequence length (bp).

Figure S3 Time-calibrated phylogenetic tree of 11 cypriniform species.

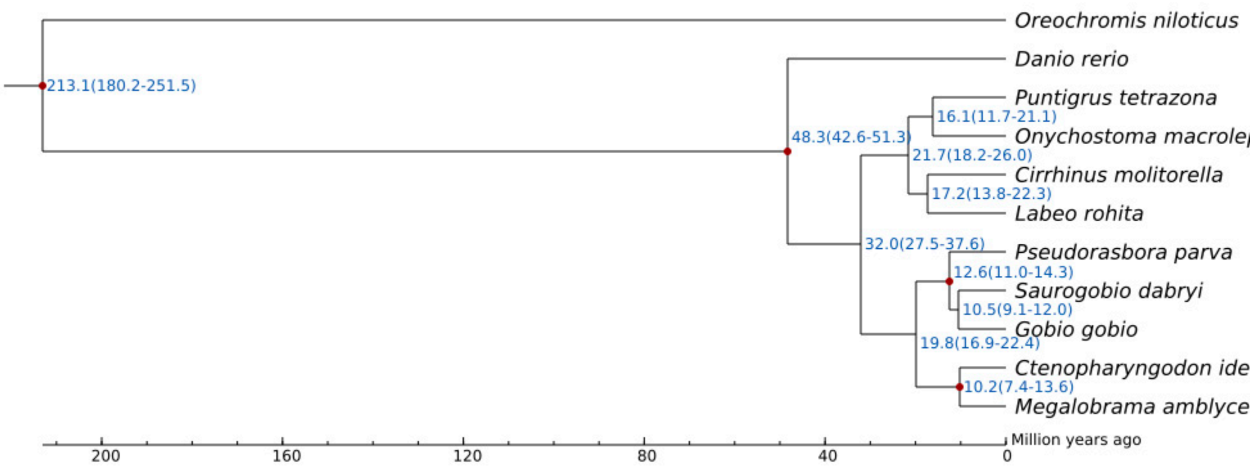

Node labels indicate divergence times in million years ago (Mya) with 95% highest posterior density (HPD) intervals in parentheses.

Figure S4 Gene family expansion and contraction across the phylogenetic tree of 11 cypriniform species.

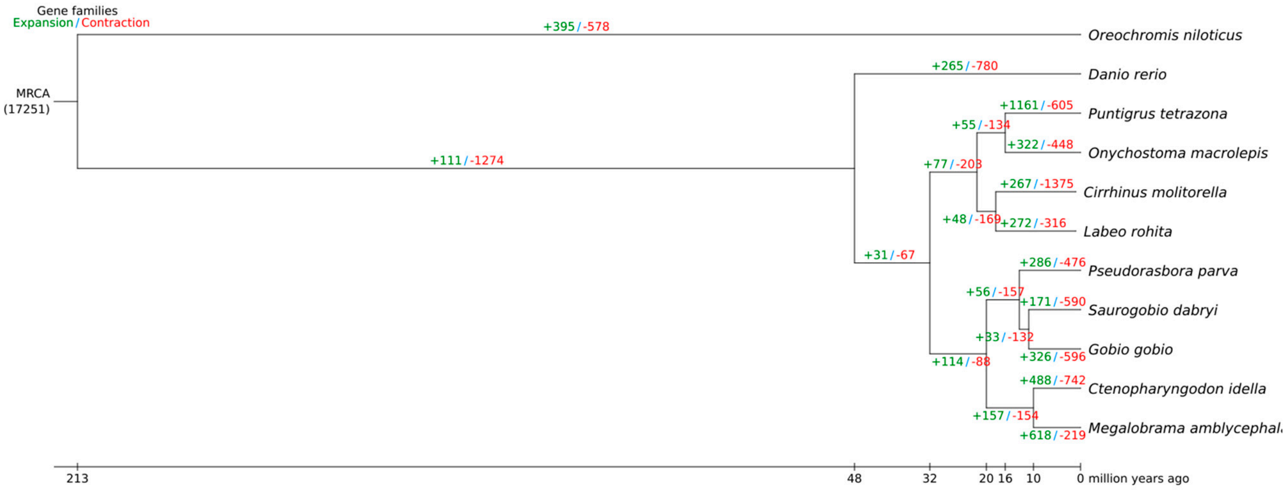

Numbers at each branch indicate the number of significantly expanded (green, +) and contracted (red, −) gene families identified by CAFE v5.0 ($P < 0.05$). The most recent common ancestor (MRCA) contained 17,251 gene families.

Figure S5 Functional enrichment analysis of positively selected genes (PSGs) in *Saurogobio dabryi*.

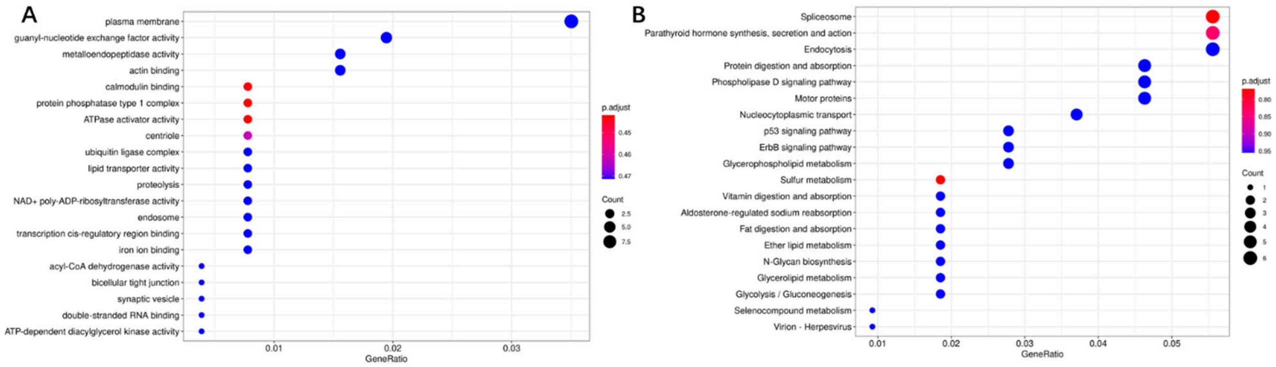

(A) Top enriched Gene Ontology (GO) terms; (B) top enriched KEGG pathways. The x-axis represents the gene ratio (proportion of PSGs annotated to a given term among all PSGs), dot size indicates the number of enriched genes, and color gradient reflects the adjusted P-value (p.adjust).
